# Advances and Current Concepts in the Medical Management of Gastroenteropancreatic Neuroendocrine Neoplasms

**DOI:** 10.1155/2017/9856140

**Published:** 2017-11-19

**Authors:** Krystallenia I. Alexandraki, Aggeliki Karapanagioti, Ioannis Karoumpalis, Georgios Boutzios, Gregory A. Kaltsas

**Affiliations:** ^1^Clinic of Endocrine Oncology, Section of Endocrinology, Department of Pathophysiology, National and Kapodistrian University of Athens Medical School, Laiko University Hospital, Athens, Greece; ^2^Department of Gastroenterology, Athens General Hospital “Georgios Gennimatas”, Athens, Greece

## Abstract

Gastroenteropancreatic neuroendocrine neoplasms (GEP-NENs) are rare and heterogeneous group of tumors presenting as localised or metastatic disease and in a subset with distinct clinical syndromes. Treatment is aimed at controlling the functional syndrome, eradicating the tumor, and/or preventing further tumor growth. Surgery is the treatment of choice in removing the primary tumor and/or reducing tumor burden but cannot be applied to all patients. Somatostatin analogs (SS-analogs) obtain control of functional syndromes in the majority of GEP-neuroendocrine tumors (NETs); phase III trials have shown that SS-analogs can be used as first-line antiproliferative treatment in patients with slow-growing GEP-NETs. The role of the recently approved serotonin inhibitor, telotristat ethyl, and gastrin receptor antagonist, netazepide, is evolving. Streptozotocin-based chemotherapy has been used for inoperable or progressing pancreatic NENs but the orally administered combination of capecitabine/temozolomide is becoming more popular due to its better tolerability and potential effect in other GEP-NENs. Phase III trials have shown efficacy of molecular targeted therapies in GEP-NETs and of radionuclide treatment in patients with midgut carcinoid tumors expressing somatostatin receptors. Most patients will develop disease progression necessitating further therapeutic options. A combination of currently available treatments along with the molecular signature of each tumor will guide future treatment.

## 1. Introduction

Gastroenteropancreatic neuroendocrine neoplasms (GEP-NENs) are considered to be rare neoplasms occurring with an incidence of 2–5/100.000 population but recent studies suggest they are more common [1]. In 2000 the WHO introduced the term “neuroendocrine” based on the immune-cytochemical demonstration of NENs for markers of neuroendocrine differentiation such as chromogranin A (CgA) and synaptophysin to denote the origin of these tumors from the diffuse endocrine system [2–4]. As GEP-NENs are composed of multipotent neuroendocrine cells they exhibit the ability to secrete bioactive substances, mainly peptides and amines, leading to distinct clinical syndromes (functioning tumors) [2]. These syndromes constitute the clinical phenotype of GEP-NENs and help making the diagnosis and monitoring response to treatment [2, 5, 6]. Nonfunctioning GEP-NENs are diagnosed following the identification of the primary tumor or through the development of metastatic disease. In their majority, GEP-NENs are slowly progressing malignancies and patients can experience prolonged survival even in the presence of metastatic disease; however, a subset may have higher proliferative activity being associated with rapid progression and poor survival [4, 7, 8].

Although GEP-NENs were initially classified according to their embryological origin it was subsequently shown that within these subgroups their clinical and biological characteristics vary considerably. This lead the European Neuroendocrine Tumor Society (ENETS) to introduce a classification system based on the anatomic rather the embryonic site of origin of GEP-NENs that reflects better their biological behavior [9, 10]. In addition, their proliferative ability was also taken into account by introducing the labeling index of the Ki-67 protein (as a mean of cell proliferation) and GEP-NENs were divided into grade 1 (G1 = Ki-67 ≤ 2%), grade 2 (G2 = Ki-67 3–20%), and grade 3 (G3 = Ki-67 > 20%) tumors [11, 12]. Grade 1 and G2 GEP-neuroendocrine tumors (NETs) are those designated to follow a rather indolent course, whereas G3 tumors exhibit an aggressive course; however, within G3 tumors those with a well-differentiated morphology have a better prognosis [13] (Table 1). These latter cases are considered a new entity classified as well-differentiated NETs G3, displaying a high proliferation index (Ki-67 index: 20%–50%) being characterized by a regular network of fine vessels, an organoid growth pattern without expansile growth, and absence of geographic necrosis or desmoplastic stroma [14]. In addition, the biological behavior of NET G3 is not as aggressive compared to NECs G3 and they exhibit a different response to treatment [14] Furthermore, the extent of the disease was also taken into consideration implementing the TNM classification system and thus allocating GEP-NENs to stages similar to other malignant neoplasms [15]. More recently, a number of genetic markers are being identified that are used to subdivide GEP-NENs further, also providing prognostic markers, and help select potential more tumor orientated treatments [16].

Another feature of GEP-NENs is that some may occur in the context of familial syndromes [2, 17, 18]. Approximately 10% are associated with the Multiple Endocrine Neoplasia- (MEN-) 1 syndrome but GEP-NENs can also be found in neurofibromatosis type 1 (NF1), Von-Hippel-Lindau (VHL) disease, and tuberous sclerosis and occasionally in familial adenomatous polyposis (FAP) [19]. The presence of a familial syndrome has to be taken into account when considering treatment options and for counseling of other family members. Due to their neuroendocrine origin GEP-NENs express on their cell surface peptide receptors, such as somatostatin receptors (sstr) that can be used for diagnostic, prognostic, and therapeutic purposes [20]. Somatostatin receptors are mostly expressed in G1 and G2 tumors (decreasing in intensity with raising Ki-67 values) and much less in G3 tumors [21]. Their expression suggests a more indolent course and is used to predict response to treatment with long acting or radiolabeled SS-analogs [22, 23].

Therapy of GEP-NENs is currently based on the intrinsic features of these tumors such as site of origin, proliferation rate (grade) and differentiation, extent of disease (stage), growth rate, and presence in the context of a familial syndrome along with the performance status of the patient. The present review will focus on established and evolving nonsurgical management of GEP-NENs briefly expanding on lung NENs that seem to respond to the same therapeutic modalities; the term carcinoid tumor will be used to denote NENs originating from the lungs. An evidence based therapeutic approach will be formulated without mentioning any advances related to surgical techniques.

## 2. Management of Functional Syndromes

A prominent characteristic of jejunal and ileal (previously named as midgut carcinoid tumors) NENs is the secretion of serotonin (5-hydroxytyramine, 5-HT) and other amines [17, 18]. Serotonin is synthesized from tryptophan and in normal subjects is used for the synthesis of nicotinic acid and in less than 1% for 5-HT [17]. However, in patients with mainly ileal NENs there is a shift towards the production of 5-HT and its metabolite 5-hydroxyindoleacetic acid (5-HIAA). In the presence of liver metastases, or when the primary lesions are found in the bronchus and/or ovaries, 5-HT is not efficiently metabolized by the liver, leading to the development of the carcinoid syndrome (CS) [17, 18]. Patients with CS may present with episodes of flushing (90%), diarrhea (70%), abdominal pain (40%), and rarely bronchospasm [24]. If CS remains untreated, it leads to a number of nutritional deficiencies, the development of carcinoid heart disease (CHD), and other fibrotic changes involving the mesentery and a worse overall prognosis [2, 25]. Early symptoms of CHD are characterized by fatigue and dyspnoea, mainly on exertion. On top of that, tumor progression which is characterised by increased serotonin levels could cause progressive right-sided heart failure leading eventually to cardiac cachexia [25].

Treatment with SS-analogs ameliorates the symptoms and long-term sequelae of CS and minimizes the risk of a carcinoid crisis that can be induced after diagnostic and therapeutic procedures and during surgery secondary to the secretion of excessive amounts of bioactive amines. Initial studies used subcutaneous (sc) octreotide administered tds (three times daily), starting at doses 50–100 mcg and increasing until a plateau dose is reached without effect on symptomatology. Doses up to 1000 mcg have been used, median dose of 450 mcg daily, obtaining a 70% control of both flushing and diarrhea along with a substantial reduction of 5-HIAA urinary levels [26, 27]. The subsequent development of long acting SS-analogs, octreotide LAR (10, 20, or 30 mg) and lanreotide autogel (60, 90, or 120 mg) administered monthly, is currently mostly employed due to their more convenient mode of administration, also obtaining a mean overall symptomatic response of approximately 65–70% of both flushing and diarrhea with minimal side effects [27, 28]. “Top-up” doses of 50 to 100 mcg (up to 1000 mcg daily) of sc octreotide can be used when symptoms are not adequately controlled with the long acting SS-analogs or in the unusual event of tachyphylaxis. Recent studies have also demonstrated that symptomatic control may also be obtained by either increasing the frequency (every two or three weeks) or the dose of long acting SS-analog administration; doses as high as 120 mg/month of octreotide LAR have been used without major toxicity [29–32]. The multireceptor targeting SS-analog pasireotide has also shown to exert at a 60 mg monthly dose similar efficacy to octreotide LAR 40 mg monthly albeit with a higher incidence of hyperglycemia (28.3% versus 5.3%, resp.) [33]. In the past interferon-*α* has also shown to be efficacious in controlling the symptoms of CS in 40–70% of patients but its use is limited due to the development of adverse effects (mainly fever, fatigue, autoimmune diseases, and myelosuppression) [2, 34]. Currently pegylated interferon has been introduced that is associated with a lower incidence of side effects whereas some older studies have suggested that the combination of long acting SS-analogs and interferon-*α* may have a synergistic effect [34–36]. Recently, the orally administered serotonin synthesis inhibitor telotristat ethyl at doses of 250–500 mg tds has been shown to provide additional symptomatic control in patients with CS [37, 38]. This agent can be used as a therapeutic option in patients with CS refractory to SS-analog administration particularly as it is associated with minimal side effects; in addition, by attenuating further 5-HIAA levels it is expected to reduce the development of peritoneal and cardiac valvular fibrosis [38]. Diarrhea may occasionally be the result of bacterial overgrowth, exocrine pancreatic insufficiency, and/or bile acid deconjugation; in such cases treatment specifically addressing these entities may be required [2]. Vitamin B supplements, to avoid niacin deficiency along with other fat-soluble vitamins, should be administered to treat other nutritional deficiencies [39].

Patients with extensive hepatic metastases and high urinary 5-HIAA levels are at increased risk of developing a carcinoid crisis [40]. Induction of anaesthesia or tumor manipulation during surgery can provoke such a crisis that is clinically presented as blood pressure alterations, particularly hypotension and rarely hypertensive crisis, prolonged and excessive flushing, hyperthermia, and bronchospasm [41, 42]. Acute intravenous octreotide provides rapid reversal of the symptoms; preoperative prophylactic treatment with intravenous octreotide infusion should be administered to patients undergoing such procedures, at an initial dose of 50 mcg/h starting 12 hours with dose titration with doses up to 500 mcg/h [40, 42, 43]. Previous treatment with long acting SS-analogs reduces but does not eliminate the risk of such a crisis to occur. In such cases, additional treatment with histamine receptor antagonists and glucocorticoids should be administered. In contrast to previous notions, inotropic support should be given in cases of intractable hypotension [42].

The most common functional pancreatic NEN (panNEN) is the insulinoma causing hypoglycemias that when diagnosed can be successfully treated with surgical resection. However, in approximately 10% of insulinomas that develop distant metastases, hypoglycemia may be refractory to conventional treatment with high intravenous glucose infusion and administration of diazoxide and SS-analogs in tumors bearing sstr2 expression [2, 44, 45]. In such cases, the multiligand SS-analog pasireotide could be an option as it causes hyperglycemia in approximately 30% of cases [46]. Recently, the mTOR inhibitor everolimus appears to exert a specific hyperglycaemic effect in metastatic insulinomas irrespective of its effect on tumor growth [44, 47, 48]. The Zollinger-Ellison syndrome (ZES) occurs as a result of gastrin hypersecretion, from duodenal and panNENs, and may be associated in up to 25% of cases with MEN-1 [49]. Proton-pump inhibitors (PPIs) obtain symptom control in almost all patients if used at appropriate high doses such as 80 mg of omeprazole [50]. Intravenous administration of 80 mg of pantoprazole tds (given 8-hourly) is efficient in obtaining rapid and prolonged control of acid hypersecretion [50, 51]. The recently synthesized orally administered gastrin receptor antagonist, netazepide, has a rapid and prolonged mode of action and could be used refractory cases when readily available [52]. Somatostatin analogs have shown to be efficacious in controlling hormonal secretion in almost all functioning panNENs including VIPomas, glucagonomas, and somatostatinomas, along with paraneoplastic syndromes related to NENs [45, 53]. It has recently been shown that the multipotent tyrosine kinase inhibitor sunitinib may exert a particular effect in VIP-secreting panNENs irrespective of its effect on tumor growth [54].

In cases where medical treatments fail to obtain adequate symptomatic relief, cytoreductive techniques including radiofrequency ablation, embolization, or chemo/radioembolization can be employed to reduce tumor, mainly hepatic, load, and the concentration of the bioactive compounds. In such cases prophylactic administration of octreotide should be given to reduce the risk of potential life-threatening crises. Particular attention should be paid in the presence of diverse clinical phenotypes secondary to the secretion of ectopically produced vasoactive compounds [53].

## 3. Management of Tumor Growth

**Figure 1:**
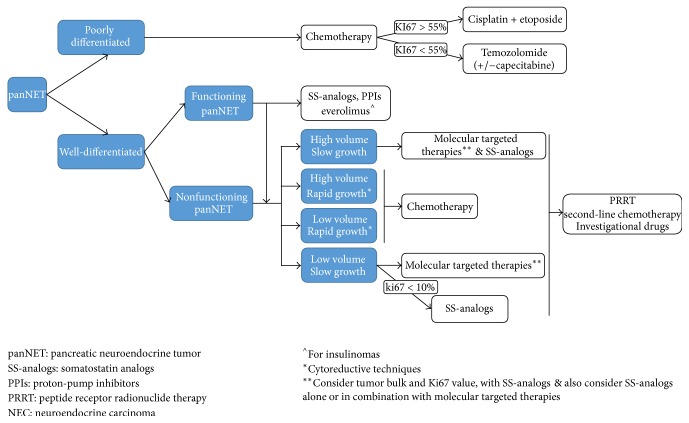
Suggested algorithm for the nonsurgical management of pancreatic neuroendocrine neoplasms based on currently available evidence.

**Figure 2:**
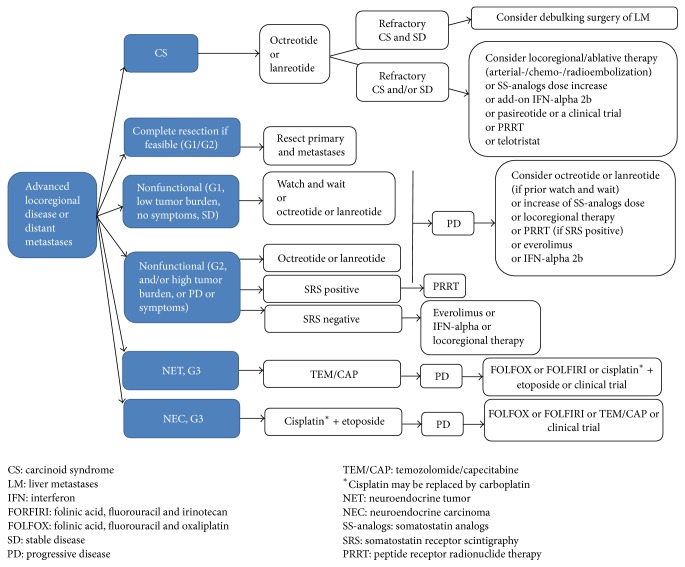
Suggested algorithm for the nonsurgical management of small bowel neuroendocrine neoplasms based on currently available evidence.

A number of therapeutic options are available for the medical management of GEP-NENs that are not amenable to surgery or they have developed disease progression including long acting SS-analogs, interferon *α*, conventional chemotherapy, molecular targeted agents, and treatment with radiopharmaceuticals known as peptide receptor radionuclide therapy (PRRT) (Figures 1 and 2) [55]. Although there is evidence to support the application of these options, there is substantial difference in currently existing level of evidence among these different modalities (Table 2).

*Long Acting Somatostatin Analogs*. Although these agents have proven to be extremely efficacious in controlling hormone secretion and the symptoms related to the majority of secretory syndromes secondary to GEP-NENs their effect on tumor growth has been a matter of debate [56]. A number of retrospective studies have previously shown that their effect was mainly cytostatic as a 5–10% response rate was obtained with the majority of studies showing disease stabilization [27, 57]. However, these studies were hampered by their retrospective nature, lack of robust criteria to access radiological responses, and information regarding prior to treatment initiation growth rate of the lesions [58–63].

Two relatively recent placebo-controlled trials (PROMID and CLARINET) have provided good quality data regarding the antiproliferative effect of these agents on GEP-NETs [64, 65]. In the PROMID study that included functioning and nonfunctioning, GEP-NETs originating from previously named midgut (carcinoid tumors) of low grade (G1) with disease progression, a prolongation of time to progression (TTP) of the octreotide versus the placebo treated group of 14.3 months versus 6 months, respectively, was obtained [64]. Responses appeared to be better in patients with low tumor burden and previously resected primary tumors [64]. In the CLARINET study that included a variety of nonfunctioning GEP-NETs of low to intermediate grade (G1 and G2 < 10%) (albeit a small number of gastric and rectal NETs were included), the progression free survival (PFS) was 32.8 months in the lanreotide compared to 18 months in the placebo treated group; the response appeared to be evident irrespective of proliferation rate, tumor load, and primary site of tumor origin [65, 66]. Although the great majority of the patients included in the study at randomization appeared to have stable disease (SD), an open label extension of the study confirmed the antiproliferative activity of lanreotide in patients of the placebo group who developed disease progression and were crossed over to open label lanreotide treatment [66]. However, there are still some unanswered questions regarding the optimal dose and duration of treatment with SS-analogs, whether treatment should be initiated only in patients with documented disease progression, what is the ideal Ki-67 cut-off associated with optimal radiological response, and whether these agents have activity in patients with G2 tumors and a Ki-67 of 10–20%. The latter question is meant to be answered by the CLARINET forte study that evaluates the response to high dose lanreotide in patients with progressive disease (PD) [67]. There is also evidence that patients with relatively low Ki-67 respond better to SS-analogs [68]. Considering the relatively prolonged PFS of patients in the placebo group in the CLARINET study, it is prudent to suggest that in patients with low tumor burden and a Ki-67 < 10%, treatment should be initiated when there is documented disease progression. However, in the presence of high tumor burden, pancreatic origin of the tumor and a relatively high Ki-67 (i.e., >5% also depending on the tissue of origin) treatment with SS-analogs could be initiated even in patients without documented disease progression. The antiproliferative effect of the more potent SS-analog, pasireotide, that has been shown efficacy of symptom control in patients resistant to conventional octreotide doses is currently formally assessed in randomized placebo-controlled trials [69]. Although a recent extended study failed to show an effect on overall survival (OS) in patients included in the PROMID study, this was merely attributed to the cross over design of the studies [70].

*Interferon*. Interferon (IFN) has been shown to exert an antiproliferative and antisecretory effect on GEP-NENs mainly through T-cell stimulation and inhibition of tumor cell-cycle progression [71, 72]. Overall objective tumor response rates of 11% along with a 50% biochemical and 75% symptomatic responses have been described [2, 6]. Subsequent studies with recombinant IFN-*α* exhibited similar response rates and substantial biochemical and symptomatic improvement [2, 6]. Although it was initially thought that the combination of SSAs and IFN-*α* had a synergistic effect, two prospective randomized studies showed an improved symptomatic control but with no further objective tumor responses [35, 36]. Current guidelines recommend the use IFN as add-on therapy to SSAs therapy in functioning tumors [8]. However, side effects with continuous IFN-*α* treatment are common including fever, myalgia, headache, fatigue, and depression and myelosuppression leading to high discontinuation rates [2, 6]. In an attempt to ameliorate these effects, pegylated IFN-*α* has been introduced and is expected to induce fewer side effects.

The antiproliferative effect of IFN-*α* was once again confirmed in a recently conducted study where patients with well-differentiated, G1/2 NETs with PD were randomized to receive octreotide LAR 20 mg every 21 days with either bevacizumab 15 mg/kg every 21 days or IFN*α*-2b 5 million units three times per week (SWOG0518) [73]. Progression free survival did not differ between the groups being 16.6 (95% CI: 12.9–19.6) months in the bevacizumab arm compared to 15.4 (95% CI: 9.6–18.6) months in the IFN arm. Currently, IFN-*β* that binds to the same receptor of IFN-*α*, but with 10-fold higher affinity is being used as it also inhibits the expression of IGF-I receptor.

## 4. Treatment with Chemotherapeutic Agents

Chemotherapy has extensively been used in the past for the treatment of GEP-NENs particularly before the availability of other medical treatments. From these mainly one arm and retrospective studies, it became apparent that well-differentiated (G1/G2) panNETs were sensitive to alkylating agents, including streptozotocin, dacarbazine, and temozolomide as well as fluoropyrimidines in contrast to GEP-NENs of other tissue origins [75]. In addition, chemotherapy remains the main therapeutic option for G3-NENs as these tumors are highly aggressive [8]. Previous studies have shown that the combination of streptozotocin and fluorouracil (5-FU) exerted a response rate of 63% compared to streptozotocin monotherapy [76]. Furthermore, the combination of streptozotocin and doxorubicin appeared to be more efficacious compared to that of streptozotocin and 5-FU, exhibiting a response rate and time to progression of 69% and 20 months compared to 45% and 6.9 months, respectively [76]. However, a large retrospective study of 84 patients with panNENs that evaluated the response rate of the combination of streptozotocin, 5-FU and doxorubicin, demonstrated a 39% response rate whereas the median response duration was 9.3 months [77]. These later findings most probably represent a more realistic figure, as the evaluation of response to therapy was performed with modern and more robust radiological means compared to the initial studies. It should be noted though that many currently used schemes do not include doxorubicin due to its cardiotoxicity and the relative good efficacy of the remaining agents of the scheme [78]. A recent retrospective study evaluated 133 patients with panNENs who were treated with the combination of STZ and 5-FU and confirmed the same response rates but identified resection of the primary and have a G3 tumor as a positive and negative predictor of OS, respectively [79]. Because streptozotocin has a relatively high toxicity profile as it can cause myelosuppression and renal impairment, alternative chemotherapeutic regimens have recently emerged. Following the findings of a phase II study that showed a 45% response rate of the combination of temozolomide and thalidomide in a small cohort of 11 patients with panNENs, a subsequent retrospective study evaluated the combination of temozolomide (oral derivative of dacarbazine) and capecitabine (oral derivative of 5-FU) in 30 chemonaive patients with panNENs obtaining a radiological response of 70% whereas the median PFS was 18 months [80–82]. Another antiangiogenic-based combination is that of temozolomide (given at 150–200 mg/m^2^ for 14 days or as metronomic daily dose) and bevacizumab exhibiting response rates ranging for 33–64% in 49 treated patients, 22 of whom had a panNEN; temozolomide has also been coadministered with everolimus exhibiting additional response rates [80]. Further studies have shown the efficacy of this regimen albeit with response rates approximately 40% [80]. Although the majority of these studies have limitations including the relatively small number of patients, differences in regimens used, and response rates obtained, temozolomide has gained popularity due to its convenient mode of administration and favourable side effect profile. In addition, recent mainly retrospective studies have shown that temozolomide based regimens may also demonstrate efficacy in GEP-NENs origin from other tissues than the pancreas [80, 83]. Currently, chemotherapy is used in patients with high tumor burden, rapidly progressive tumors and when the Ki-67 labeling index is relatively high [8].

The role of chemotherapy is also well established in patients with neuroendocrine carcinomas (NECs). These high-grade malignancies are locally advanced or metastatic at presentation, only rarely express sstrs, and are usually not associated with a secretory syndrome. First-line systemic chemotherapy with a platinum based agent (cisplatin or carboplatin) and etoposide is recommended for most patients with metastatic-stage disease, whereas sequential or concurrent chemoradiation is recommended for patients with locoregional disease [84]. Response rates ranging from 42 to 67% have been described but are usually of short duration whereas the median survival ranges from 15 to 19 months [84]. Based on the response, usually 3-4 of cycles of chemotherapy are administered but following relapse or nonresponse second-line options are limited [84]. A recent retrospective study has suggested that patients with panNECs and ki-67 values of less than 55% may respond better to temozolomide based regimens compared to cisplatin combination [85–87]. Hence, the new classification of G3 NETS may be helpful to direct the more appropriate chemotherapeutic scheme [85]. For patients that have failed to respond or have developed progression a number of second-line treatments have been tried but the response rates are low and patients develop PD.

## 5. Therapy with Radiolabeled Peptides

The basic principle of applying treatment with radiopharmaceuticals in GEP-NETs relies on the use of high-energy *β*-emitters to generate radiation-induced DNA damage in targeted somatostatin receptor (sstr) expressing GEP-NETs [2, 88]. Several radioisotopes have been used including Indium-111, Yttrium-90, and Lutetium-177 (a combined *β* and *γ* emitter) and the response relies on the density of expression of sstr on the cell tumor surface [89]. A large single center including 310 patients using ^177^Lu-Dotatate showed a complete or partial response in 2% and 28% of patients, respectively, with a significant number of patients demonstrating SD [89]. In addition, the median time to progression was 40 months and median OS from the start of treatment was 46 months. Another radiopharmaceutical, 90-Yttrium-edotreotide was used in 90 patients with metastatic CS and symptoms refractory to octreotide and showed durable responses, with PFS significantly greater in patients with sustained improvement in diarrhea, and with an acceptable expected adverse effect profile after 3 cycles of 4.4 GBq of 90Y-edotreotide every 6 weeks [90]. A meta-analysis of many patients showed that side effects of this treatment were relatively minor [91]. As most of these data were derived from single center and retrospective studies a subsequent phase III multicentre study evaluated the efficacy of 177Lu-Dotatate in midgut NENs with documented PD (NETTER-1) [92]. Patients receiving this treatment were compared to patients on high dose administration of octreotide (60 mg/month) [92]. The estimated rate of PFS at month 20 was found 65.2% (95% confidence interval [CI], 50.0–76.8) in the 177Lu-Dotatate group, and 10.8% (95% CI, 3.5–23.0) in the control group, while the median PFS had not yet been reached in the 177Lu-Dotatate group and was 8.4 months (95% CI, 5.8 to 9.1) in the control group (hazard ratio [HR] for PD or death with 177Lu-Dotatate versus control, 0.21; 95% CI, 0.13 to 0.33; *P* < 0,001), which represented a 79% lower risk of PD or death in the 177Lu-Dotatate group than in the control group. Regarding the OS analysis, a risk of death 60% lower in the 177Lu-Dotatate group than in the control group was estimated (HR for death with 177Lu-Dotatate group versus control, 0.40; *P* = 0.004) but the data were not sufficiently mature to provide an estimate of the median OS in either treatment group. Finally, when the tumor response was evaluated, the total number of complete and partial response in the 177Lu-Dotatate group (18 patients) and the control group (3 patients) corresponded to response rates (RR) of 18% and 3%, respectively (*P* < 0.001). Hence, the data obtained from this later study suggest that PRRT is a very efficacious treatment associated with the best PFS and response rates obtained up to now compared to other currently available therapies. Recent evidence suggests that the response to treatment can be predicted by the concomitant uptake of the lesions of 18FDG-PET [93]. In addition, it is believed that the introduction of molecules that have a higher affinity to octreotide may enhance the therapeutic effect [92].

## 6. Molecular Targeted Therapies

As several pathways of intracellular signal transduction have been found to be active in patients with GEP-NENs a number of agents inhibiting these pathways have lately been evaluated in a number of phase III trials. Components of the mTOR pathway have been shown to be activated in GEP-NENs, more so in panNENs than in other GEP-NENs [94]. RAD001 (everolimus) is an oral inhibitor of mTOR first investigated in a phase II study in 30 patients with carcinoid and 30 with panNETs in combination with octreotide LAR 30 mg every 4 weeks showing efficacy [95]. A further open label phase II nonrandomized study (RAD001 in Advanced Neuroendocrine Tumors (RADIANT 1)) showed that GEP-NET patients with PD treated with 10 mg of everolimus plus octreotide LAR 30 mg obtained a 4.4% partial response, 80% disease stabilization, and a median PFS of 16.7 [96]. These findings led to a number of phase III studies that evaluated in a prospective manner the efficacy and side effect profile of this agent in GEP-NETs of different primary origin. The RADIANT 3 study evaluated 207 patients with advanced low- or intermediate-grade panNETs with radiological progression within the previous 12 months to receive 10 mg of everolimus along with best supportive treatment compared to 203 patients who received placebo [97, 98]. The median PFS was 11 months with everolimus compared to 5.6 months to placebo (HR 0.35), with relatively minor side effects mainly rash, diarrhea, fatigue, and upper respiratory tract infections; grade 3-4 events were anemia and hyperglycemia. RADIANT 4 trial evaluated the efficacy of everolimus in nonfunctioning progressive intestinal (other than panNETs) and lung NETs in a similar to RADIANT 3 design and showed a median PFS of 11 months with everolimus compared to 3.9 months of placebo [99]. On the contrary, the efficacy of this agent along with octreotide was less evident in patients with midgut NETs and CS [100]. However, treatment is not without side effects including among others stomatitis, fatigue, and diarrhea necessitating dose reduction in a substantial number whereas 12–19% of patients withdrew from the studies [96–100]. On the basis of the promising activity of everolimus, several phase II studies combinations with temozolomide, sorafenib, bevacizumab, and pasireotide are underway (https://www.clinicaltrials.gov).

Angiogenesis plays an important role in the development of NENs [95]. Well-differentiated GEP-NETs express higher levels of Vascular Endothelial Growth Factor (VEGF) and platelet-derived growth factor (PDGF) and higher microvessel density, making them potential targets for medications that inhibit these pathways. Sunitinib is a TKI that inhibits VEGF1–3, PDGF, stem-cell factor, glial cell line-derived neurotrophic factor, and FMS-like tyrosine kinase-3 (Flt 3) receptors and exhibits antiangiogenic and antiproliferative activity. Based on one partial response observed in 4 patients with NETs included in a phase I study, sunitinib was evaluated in 107 patients with well-differentiated GEP-NETs. Patients with panNETs experienced a partial response of 16.7% whereas patients with other GEP-NETs a 2.4% response albeit a high rate of SD was found in both tumor types [95]. Based on these promising preliminary results, a multicentre phase III study of sunitinib (37.5 mg/d) versus placebo was performed in patients with progressive panNEΤs obtaining a PFS of 11.4 months compared to 5.5 months in the control group [HR 0.42, 95%-CI 0.26–0.66, *P* < 0.001]. The most commonly reported grade 3 and 4 adverse effects in treated patients were neutropenia, hypertension, hand-foot syndrome, abdominal pain, diarrhea, and fatigue [101]. A number of other TKI involving mainly phase II studies and including a smaller number of patients have produced less consistent results [95].

Recently the effect of these agents on OS has been assessed. Although no definite benefit has been documented this was most probably attributed to the cross over design of the studies [96, 102].

## 7. Other Drugs Targeting VEGF and Evolving Treatments

Bevacizumab is a monoclonal antibody directed against VEGF [103, 104]. A randomized phase III trial compared octreotide plus IFN*α*-2*β* or bevacizumab in advanced GEP-NENs demonstrating similar efficacy and suggesting that bevacizumab could be incorporated into future trials of advanced GEP-NENs [73]. In addition, a phase II study investigated that bevacizumab and temozolomide combination in advanced panNENs showed a 24% partial response [95, 104], while the combination of everolimus and bevacizumab was shown to be well tolerated exhibiting a 26% response rate in patients with advanced NENs [104, 105].

However, there are not enough data regarding the use of other MTTs, such as sorafenib, pazopanib, or axitinib in non-panNENs or in panNENS. These compounds, such as bevacizumab and sunitinib, are studied in midgut NENs (SUNLAND study: a study of sunitinib versus placebo in combination with lanreotide in patients with progressive advanced/metastatic midgut carcinoid tumors) and in other prospective clinical trials (https://www.clinicaltrials.gov).

Recently immunotherapy has been investigated in a number of malignancies exhibiting substantial results in patients with melanomas and some tumors of neuroendocrine origin such as Merkel cell carcinoma [106, 107]. Up to now only few data are available for these agents in NENs [108]. It is intended by ENETS to evaluate these agents particularly in G3 tumors. Besides the availability of a number of agents, drugable genomic alterations are low in GEP-NENs. It is expected that the identification of specific molecular alterations may expand currently available therapeutic options allowing a personalised approach.

## 8. Conclusions

The majority of patients with GEP-NENs are well-differentiated tumors and exhibit a relatively favourable prognosis besides extensive disease. Patients with functional syndromes and extensive disease can be effectively treated with long acting SS-analogs whereas specific functional syndromes may respond to PPIs, everolimus, and telotristat ethyl. SS-analogs constitute the first-line antiproliferative treatment in G1 and G2 tumors with PD whereas upon further progression a number of options are available based on patients' characteristics including molecular targeted therapies, chemotherapy, and PRRT. Chemotherapy is preferred in panNENs, high tumor burden, and fast-growing tumors whereas PRRT is preferred in patients with high avidity to SRS. Selection of treatment is best scheduled by a multidisciplinary team in order to have a centralization of their database that may result in periodical audit of their diagnostic procedures and therapeutic modalities.

## Figures and Tables

**Table 1:** Novel pathological classification suggested.

	G1 NETS	G2 NETS	G3 NETS	G3 NEC
Ki-67 index (% of positive cells per 100 counted cells)	<2	2–20	>20 (20–50)	>20 (>50)
Mitotic count (number of mitoses per 10 high-power field)	<2	2–20	>20	>20
Morphology	Well-differentiated	Well-differentiated	Well-differentiated	Poorly differentiated

NETS: neuroendocrine tumors.

**Table 2:** Factors that need to be considered in order to select the most appropriate treatment along with currently available nonsurgical treatments for pancreatic neuroendocrine neoplasms.

*Tumor-related factors*		
Functioning tumor	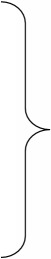	SS-analogs Chemotherapy Molecular targeted therapy Liver-directed therapy PRRT
Grading		
Extent of disease (liver or other metastases)		
Extent of liver involvement		
Tumor growth rate		
SRS uptake		
*Patient-related factors*		
Patient performance status		
Presence of a familial syndrome		
Patient's preference		
*Health-economy-related factors*		
Local availability		

SRS: somatostatin receptor scintigraphy; SS: somatostatin; PRRT: peptide receptor radionuclide therapy.
